# From Aerosol to Signal: Advances in Biosensor Technologies for Airborne Biothreat Detection

**DOI:** 10.3390/bios15120764

**Published:** 2025-11-21

**Authors:** Samuel De Penning, Md Sadiqul Islam, Kawkab Ahasan, Todd A. Kingston, Pranav Shrotriya

**Affiliations:** Center for Multiphase Flow Research and Education, Department of Mechanical Engineering, Iowa State University, Ames, IA 50011, USA; samdp@iastate.edu (S.D.P.); sadiqul@iastate.edu (M.S.I.); kahasan@iastate.edu (K.A.); kingston@iastate.edu (T.A.K.)

**Keywords:** aptasensors, electrochemical biosensor, airborne particle capture, microfluidics, condensation-based growth, biothreat detection

## Abstract

The growing threat of airborne biological agents necessitates rapid, sensitive, and portable detection systems to mitigate risks to public health and national security. We present a comprehensive overview of biosensor technologies developed for airborne biothreat detection, with a focus on aptamer-based electrochemical sensors. These sensors offer key advantages in portability, chemical stability, and adaptability for multiplexed detection in field settings. The urgency for real-time surveillance tools capable of identifying viral, bacterial, and toxin-based agents is discussed, particularly in the context of biodefense. Aerosolized particle capture strategies are reviewed, focusing on microfluidics for micron-sized particles and condensation-based systems for submicron-sized particles, which are preferred for their small-volume operation and seamless integration with biosensors. Key biosensor components are described, including recognition elements—such as aptamers—and transduction mechanisms like electrochemical impedance spectroscopy. EIS is highlighted for its label-free, miniaturizable, and real-time readout capabilities, making it well-suited for portable biosensors. Advances in sensing strategies for both viral and bacterial targets are explored, featuring innovations in nanoporous membrane platforms, nanomaterials, and multiplexed assay formats. Recent developments demonstrate improved sensitivity through nanopore-based signal amplification and enhanced selectivity using engineered aptamer libraries. The review concludes by addressing current limitations, including environmental stability, system integration, and the need for validation with complex real-world samples. Future directions point toward the development of fully integrated, field-deployable biosensing platforms that combine effective aerosol capture with robust and selective biosensing technologies.

## 1. Introduction

The detection of airborne biothreats is a critical concern for public health and national security. Biothreats have the potential to cause widespread disease outbreaks, and their detection requires rapid, accurate, and reliable methods [1]. As such, significant research has been dedicated to developing advanced technologies that can detect these pathogens in real-time, with minimal sample preparation and high sensitivity. Among these technologies, biosensors have emerged as promising tools for detecting biological agents due to their ability to provide fast, sensitive, and cost-effective solutions [2,3].

Biosensors are analytical devices that utilize a biological recognition element to detect the presence of target molecules [4]. These sensors have the capability of being configured for the specific detection of several relevant biothreats. However, a key challenge in developing biosensors for the detection of airborne biothreats is the efficient collection of aerosols from the environment. Many dangerous diseases and biothreats are present in the form of aerosols, or can be deliberately aerosolized, and pose a significant risk of airborne transmission [5], such as *Bacillus anthracis* (Anthrax), SARS-CoV-2, and Viral Hemorrhagic Fevers (e.g., Marburg virus, Ebola virus), to name a few [6,7,8]. These airborne pathogens are typically present in extremely low concentrations (<100 particles/m^3^), and successfully capturing them is an essential prerequisite for accurate detection [9]. Microfluidics has emerged as a powerful tool in addressing this challenge. Microfluidic systems can capture and enrich airborne particles, facilitating the concentration of biothreat samples for subsequent analysis [10]. When combined with biosensors, microfluidics enables real-time, continuous air quality monitoring and pathogen detection. This combination of microfluidic-based particle capture and biosensing offers a promising approach for developing next-generation biosensors that respond rapidly and efficiently to airborne biothreats.

**Figure 1:**
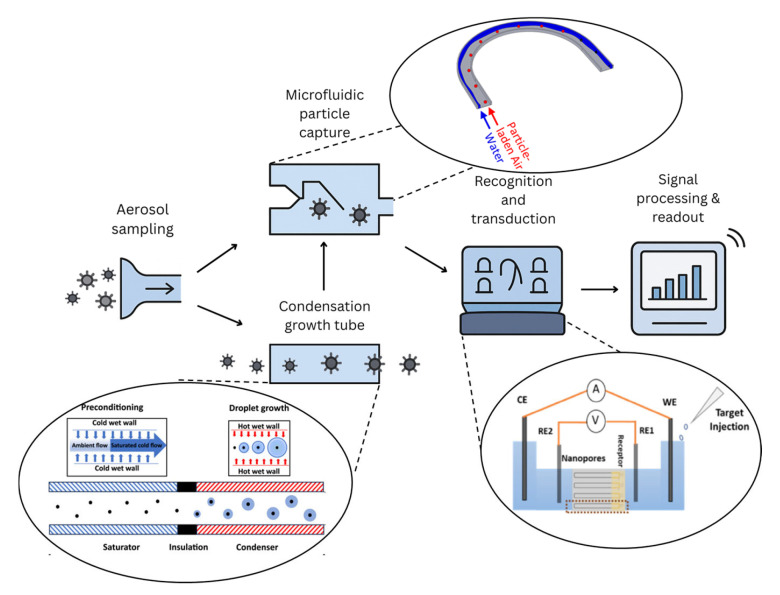
Schematic overview of the integrated detection pipeline for airborne biothreats. Airborne pathogens are first collected via aerosol sampling and optionally enhanced through condensation-based particle growth. Particles are then captured and concentrated using a microfluidic system, followed by recognition and signal transduction through the biosensor. Finally, the signal is processed and read out, providing a real-time detection output. Portions of this figure are adapted from Refs. [11,12,13].

In this review, we critically examine multiple approaches to particle capture, including microfluidic systems for submicron particles (e.g., certain bacteria and fungal spores) and condensation-based methods for nanometer-sized particles (e.g., viruses). We explore the current state of biosensor technologies for the detection of biothreats and the integration of microfluidic-based aerosol capture. We examine various biosensor configurations and their uses for biothreat detection. We also discuss the challenges in optimizing these systems for real-time, high-efficiency biothreat detection and the recent advancements that have made significant strides toward overcoming these limitations. The complete detection pipeline, from airborne pathogen capture to biosensor signal readout, is illustrated in Figure 1, providing a visual overview of the integrated workflow that underpins the subsequent sections of this review.

## 2. Biothreats and Their History

Biothreats, also known as bioagents, are pathogens and toxins of biological origin that pose significant risks to human, animal, and environmental health [14]. These threats encompass a broad spectrum of viruses, bacteria, fungi, and toxins, which vary widely in transmissibility, infectivity, detectability, and lethality [14]. This diversity underscores their complex and multifaceted nature, with some capable of rapid human-to-human transmission and global spread. A pandemic, an epidemic that crosses international boundaries and affects large populations, is one of the most severe consequences of biothreats [15]. Throughout history, pandemics have left profound and lasting impacts on societies, economies, and geopolitical stability. A historical overview of major epidemics and pandemics is presented in Table 1.

Among these pandemics, the Black Death stands as one of the deadliest, killing an estimated 75–200 million people across Europe and Asia between 1346 and 1353 [19]. Smallpox, which may have been prevalent since 10,000 B.C., claimed approximately 300 million lives since 1900 before being eradicated through global vaccination efforts [20]. In more recent times, diseases such as influenza, HIV/AIDS, and COVID-19 continue to demonstrate the constant threat posed by air-transmitted pathogens. Globally, infectious diseases are responsible for an estimated 15 million deaths annually [21].

### Biowarfare and Bioterrorism

In addition to natural outbreaks, biothreats have been deliberately weaponized throughout history. Bioterrorism refers to the intentional release of pathogens or biological toxins to harm civilian populations, while biowarfare involves the use of such agents against military targets [22,23]. Early biowarfare tactics included contaminating weapons and water supplies with pathogens. A few early historical examples would be Emperor Fredrick Barbarossa’s poisoning of enemy wells with corpses and the catapulting of plague-infected corpses into besieged cities in medieval times [24].

Scientific advancement further accelerated the development of biological weapons in the 20th century. In World War I, Germany deployed *Bacillus anthracis* (anthrax) and *Burkholderia mallei* (glanders) to infect livestock and disrupt enemy supply chains [24]. World War II saw further developments, including Japan’s Unit 731, which released *Yersinia pestis*, *Salmonella Typhi* (typhoid), *Vibrio cholera* (cholera), and *Bacillus anthracis* in China through aerial dispersal and infected vectors [25,26].

In the modern era, non-state actors such as terrorist organizations and rogue groups have emerged as major threats in the realm of biological weapons [27]. A notable example is the 1984 Rajneesh cult attack in Oregon, where *Salmonella typhimurium* was deliberately spread via contaminated salad bars [6]. Similarly, the 2001 anthrax attacks in the U.S.—occurring shortly after the September 11 terrorist attacks—demonstrated the disproportionate psychological and political impact of biological terrorism [6,28].

To help prioritize response efforts, the U.S. Centers for Disease Control and Prevention (CDC) categorizes biological agents into three priority groups based on their threat level [24]. Category A agents are considered the highest priority and include pathogens that pose a significant risk to national security due to their ease of transmission, high mortality rates, and potential to cause public panic and social disruption. Category B agents are the second-highest priority and include moderately easy-to-disseminate pathogens that result in lower mortality rates but still require enhanced surveillance and diagnostic capacity. Category C agents include emerging infectious diseases that could be engineered for mass dissemination in the future due to their availability, ease of production, and potential for high morbidity and mortality. Table 2 presents these categories, focusing on Categories A and B.

Despite significant advancements in medical treatments and containment strategies, early detection of biological threats remains critical for outbreak prevention and control. Many biological agents can spread insidiously, often remaining undetected until they have already achieved widespread transmission. Consequently, developing and deploying rapid, sensitive, and reliable pathogen detection technologies is essential for enabling timely responses, guiding post-exposure interventions, and mitigating the public health and security impacts of both natural and deliberate biological events. Since many pathogens of concern are airborne, transmitted through aerosols or fine particulates, they must first be efficiently captured from the air before any sensing or analysis can occur. Effective preconcentration methods, such as microfluidic-based particle capture and condensation growth techniques, are thus foundational in enabling accurate biosensing of airborne biothreats.

## 3. Particle Capture

### 3.1. Microfluidic-Based Capture

Microfluidics is a field that focuses on the manipulation and control of fluids within sub-millimeter-scale structures, typically involving channels ranging from tens to hundreds of micrometers in width [29,30,31]. These small-scale systems offer several advantages over conventional fluidic platforms, including reduced reagent consumption, faster analysis time, and higher surface area-to-volume ratios that enhance reaction kinetics [32,33]. Beyond these general benefits, microfluidic platforms are particularly well suited for airborne biothreat detection because they operate at small fluid volumes, enable precise handling of scarce samples, and can be directly integrated with biosensor systems. Hence, in this review we primarily limit our discussion of particle capture to microfluidic system. Consequently, microfluidics has been widely adopted for rapid biosample processing and has shown promise in real-time, continuous collection and enrichment of airborne biothreat agents for delivery to on-chip biosensors [34,35,36].

In recent years, microfluidic studies on biothreat detection have focused on cell separation, enrichment, and detection using liquid media [37,38,39,40,41,42,43,44], with only a few focusing on biothreat detection from aerosols. Among the studies on aerosol detection, microfluidic architectures have been explored for aerosol capture, employing designs such as curved channels, herringbone structures, spiral paths, and impaction-based mechanisms. For example, staggered herringbone microchannels have been used to capture aerosolized *Escherichia coli* (used as a model organism), *Mycobacterium smegmatis*, and *Mycobacterium tuberculosis* [45,46,47,48,49]. Li et al. [50] extended this concept to the detection of airborne fungal spores such as *Aspergillus niger*. Similarly, Bian et al. [51] developed a three-loop spiral microchannel with herringbone and sawtooth structures to enhance mixing and particle capture. Inami et al. [52] developed a semi-automated microfluidic chip system that integrated aerosol collection, spore treatment, and isothermal DNA amplification (ICAN). Shen et al. [53] introduced a silicon nanowire field-effect transistor integrated with a microfluidic system for real-time monitoring of biological aerosols, including the influenza virus, but their device exhibited limited sensitivity, with only a 20–30% signal increase even after a ten-fold increase in virus concentration. These devices generally demonstrated capture times between one and three hours and often required multiple manual steps, limiting their real-time applicability.

To address the limitations of throughput and sensitivity, researchers have investigated several physical mechanisms to enhance particle capture. Mirzaee et al. [54] utilized inertial impaction in a microfluidic on-chip impinger. Kang et al. [55] applied inertial forces in conjunction with mini-fluorescent microscopy to detect microorganisms in real-time. Damit [56] introduced a droplet-based microfluidic device coupled with a wet-cyclone sampler for particles between 2–5 µm in size, but their approach showed poor sampling efficiency for submicron particles. Hong et al. [57] introduced a microfluidic system capable of separating submicron particles based on size, but the particle capture efficiency was limited to 70% and involved complex operation. Ma et al. [58] designed an electrostatic microfluidic sampler that achieved only 40% efficiency for particles smaller than 5 µm. However, electrostatic sorting can significantly alter the biological properties of collected microorganisms [59]. Liu et al. [48] presented a microfilter-based PDMS membrane device that was low-throughput and time-intensive. One of the more promising advances was made by Choi et al. [60], who developed an inertial-based aerosol collection and enrichment system. Their device used a U-shaped channel with a stratified liquid stream to transfer aerosolized particles between 0.6–2.1 µm into the liquid phase. This system improved capture efficiency and avoided the limitations observed in prior technologies, such as low throughput and poor submicron performance. Ahasan et al. [11] numerically and experimentally investigated a similar stratified flow-based U-shaped microchannel and introduced two distinct performance metrics—entrapment efficiency and diversion efficiency—to differentiate whether or not the particles were being collected into the liquid stream. Table 3 summarizes and compares different microfluidic-based capture systems.

**Figure 2:**
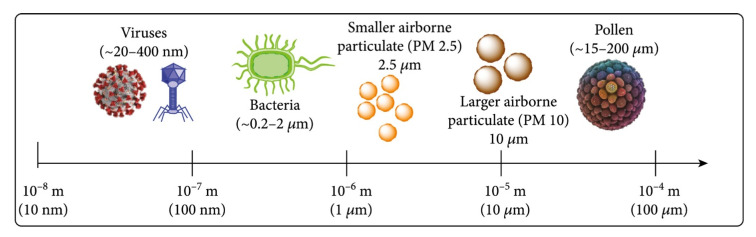
Size ranges of various types of microorganisms, including viruses, bacteria, and other microbial entities. This comparison highlights the vast diversity in biothreat dimensions, which is critical for designing effective collection and detection systems. Reprinted from Ref. [61].

Although these systems demonstrate significant progress in real-time capture and detection of airborne biothreats, several challenges remain. As illustrated in Figure 2, airborne particles span a broad size range—from nanometer-scale viruses to micrometer-scale pollen—which presents a significant challenge to achieving both efficient and real-time capture of all particle sizes. Most devices show reduced performance for submicron particles like viruses, often involve complex fabrication or multistep procedures, with requirements of subsequent manual steps, and/or rely on bulky instrumentation, which complicates field deployment, for detection of captured biothreats. Another critical limitation is that many collection devices separate/enrich the aerosol while suspended in the air. Doing so often requires further processing to transfer them into liquid media to enable electrochemical sensing.

### 3.2. Condensation-Based Growth Tube for Submicron Particles

One of the significant limitations of any inertial-based capture device is the sharp decline in capture efficiency with decreasing particle diameter [11,60]. Biothreat agents consist primarily of two major classes of microorganisms: bacteria and viruses. While airborne bacterial particles typically fall within the micron range, viruses are significantly smaller, ranging from approximately 20 to 400 nm [62,63]. For example, SARS-CoV-1 and SARS-CoV-2 virions exhibit diameters between approximately 50 and 200 nm [64]. Influenza A viral particles have an average diameter of around 120 nm. Respiratory syncytial virus (RSV) particles are filamentous, with diameters near 130 nm [61,65,66]. Measles virus particles measure approximately 100 to 200 nm in diameter [67]. Mumps virus particles show a broader size distribution, ranging from 100 to 600 nm [68].

While bacteria lie within the ideal capture size range for inertial microfluidic devices, viruses tend to follow the airflow streamlines due to their low inertia, eluding capture [69]. However, these submicron particles can act as nucleation sites for water-vapor condensation. When submicron particles pass through a supersaturated environment, such as a condensation-based particle growth tube, due to heterogeneous condensation, the particles are encapsulated in a liquid layer and increase their overall size. A similar phenomenon naturally occurs in the human respiratory tract, which acts like a natural particle growth tube. Upon entering the respiratory tract, aerosol particles encounter regions of high humidity and supersaturation, especially in the upper airways, where water vapor supersaturation can promote condensation-based particle growth on inhaled particles. This process contributes to the deposition and clearance of inhaled aerosols and has been extensively studied in the context of respiratory drug delivery and environmental exposure [70].

Quantifying submicron particles is inherently challenging due to their size falling below the resolution limits of many standard measurement techniques [71]. Condensational growth has long been employed to overcome this limitation by encapsulating the particles thereby making them more easily detectable [72]. Heterogeneous condensation plays a central role in this approach and involves vapor condensing on pre-existing particles, in contrast to homogeneous condensation, which occurs in the absence of such nuclei. Classical studies on adiabatic expansion have laid the groundwork for understanding the role of heterogeneous condensation in encapsulating airborne particles [73]. The process of heterogeneous condensation on a solid, insoluble submicron-sized particle is not energetically favorable because the large surface area-to-volume ratio of small particles leads to a rise in free energy barrier for condensation [74]. The vapor must be supersaturated to surpass the energetic barrier and initiate condensational growth [75]. Thomson [76] (also known as Lord Kelvin) first theoretically demonstrated that the smaller the particles, the higher the supersaturation required to initiate condensational growth. This phenomenon occurs because the equilibrium vapor pressure over a droplet is higher than over a flat surface due to the surface curvature. The expression for the minimum diameter, which is commonly referred to as the Kelvin diameter, required for condensation relates supersaturation and surface tension [77]. It is important to note that while this equation was originally formulated to characterize homogeneous droplet formation, it can also be applied to describe liquid embryos developing on surfaces [78].

**Figure 3:**
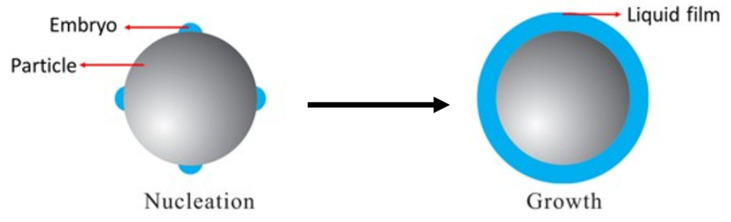
Schematic of heterogeneous condensation on a spherical particle. Source: Adapted from Ref. [79].

The encapsulation of particles in water through heterogeneous condensation occurs in two stages: a nucleation stage and a growth stage. Initially, a liquid embryo nucleates on the particle surface. Subsequently, the vapor condenses around one or more embryos, resulting in encapsulation of the particle in a liquid layer that grows in thickness; this is known as the growth stage. The heterogeneous condensation process is schematically shown in Figure 3.

Although particle encapsulation via heterogeneous condensation using ethanol or butanol has been established for over a century, the development of water-based condensation devices represents a relatively recent advancement [80,81,82]. These systems are increasingly favored for their compatibility with clean environments and their suitability for bioaerosol collection and analysis. Hering and Stolzenburg [83] developed an innovative approach for increasing particle size using water vapor condensation in a laminar, thermally diffusive flow. Their system introduces airflow into a wet-walled tube, where the wall temperature is higher than the inlet flow, creating a condensation-based growth tube comprising two key sections: the conditioner and the initiator. The conditioner reduces the temperature and increases the relative humidity of the incoming air, while the initiator—which is heated relative to the conditioner—facilitates particle nucleation and growth. Hering and Stolzenburg [83] demonstrated that the temperature difference between these two regions influences nucleation efficiency (i.e., the fraction of particles that become nucleated and subsequently grow via condensation). The mass diffusivity of water vapor is greater than the thermal diffusivity of air, resulting in a faster water vapor flux to the centerline than the heat flux from the walls. Additionally, Hering et al. conducted a comparative study on detection efficiencies and response times for organic versus inorganic particles, revealing that particles as small as 4.8 nm could be detected with 50% efficiency, though organic particles required larger sizes (8–30 nm) depending on compound purity [84]. Lewis and Hering [85] further explored how particle concentration affects condensation growth. Their results revealed that particle concentration greater than 10^5^ particles/cm^3^ leads to a decrease in the saturation ratio. The reduction in flow supersaturation is due to water vapor depletion by droplet absorption and an increase in equilibrium vapor pressure from the heat released during condensation.

Expanding on this foundation, Hering et al. [86] later introduced a “moderated” laminar-flow water-condensation method which enables the attainment of the same peak supersaturation and droplet growth while minimizing the addition of heat and water vapor. Hering et al. [87] later developed a water-based condensation particle counter (WCPC) capable of detecting particles down to ~1 nm by evaluating ion detection and time response performance. Additionally, Hering et al. [88] also developed a motion-tolerant, self-sustaining WCPC featuring an improved moderator stage. Oh et al. [89] introduced modified bio-sampler designs to improve particle collection, which were then used to demonstrate the effective collection of *MS-2 viruses* [90,91], *S. kudriavzevii yeast*, *E. coli* [92], and *Influenza virus* [93] using condensation-based devices, also examining factors such as virus viability and nebulizer concentration. Lee et al. [94] developed a microfluidic-based biosensor integrating condensation for real-time ATP detection from airborne microbes. Yu et al. [95] investigated heterogeneous condensation within a two-stage growth tube to study the growth of SiO_2_ particles from coal combustion. Numerical and experimental studies by Tammaro [96], Xu et al. [97], Bian et al. [13], and Yu et al. [98,99] have demonstrated the ability to enhance nanoparticle capture from combustion sources through careful modulation of supersaturation profiles. Notably, Kwon et al. [100,101] designed a micro-electro-mechanical system-based condensation particle counter with a microfabricated wicking structure, allowing for the detection of particles as small as ~9 nm. Balendra et al. [102] and Yu et al. [98] analyzed the physical and parametric constraints involved in miniaturizing these devices using non-dimensional models and semi-empirical methods. Furthermore, Yoo et al. [103] presented a microfluidic condensation bioaerosol sampler (MCBS) that integrates a virus growth section and droplet-to-liquid conversion section on a chip. Ahasan et al. [74] used molecular dynamics to show that higher glycoprotein density on viral envelopes enhances condensation in humid environments, such as growth tubes. Chen et al. performed a quantitative analysis of the deviation in detection efficiency of aviation soot relative to calibration particles in a condensation particle counter (CPC) [104]. Li et al. developed a convertible CPC that can operate with either alcohol or water as the working fluid, without altering the instrument structure or wick material [105]. Using water, they reported cutoff diameters of 3 nm for NaCl particles and 2.8 nm for Ag particles. In a separate study, Chen et al. enhanced the condensation-based growth device by integrating particle steam agglomeration, which increased both agglomeration and particle separation efficiencies [106]. Dai et al. investigated particle growth in turbulent water-vapor environments and demonstrated that soluble and hydrophilic particles in atmospheric fine particulate matter exhibit greater growth than coal particles [107]. Moreover, the particle growth tube with heterogeneous nucleation technology is particularly notable for its high efficiency, low energy consumption, and absence of secondary pollution, making it a subject of significant attention and research [108,109]. These findings collectively demonstrate that condensation-based particle growth is not only a robust strategy for overcoming the lower capture limit of inertia-based devices but also a promising avenue for enhancing bioaerosol detection sensitivity and selectivity.

In summary, water-based condensation growth tubes utilize controlled supersaturation to encapsulate submicron particles, greatly improving capture and detection efficiency. By precisely managing temperature and humidity profiles, these systems effectively promote particle nucleation and growth. Integrating condensation growth tubes with microfluidic platforms offers a promising strategy to enhance nanoparticle collection and enable real-time detection of bioparticles with high sensitivity.

## 4. Biosensors

Rapid and reliable pathogen detection is critical for improving biothreat monitoring, controlling disease spread, and enabling faster treatment, ultimately saving lives. Biosensors are receiving extensive research attention due to their potential for rapid, multiplexed, and accurate detection of biothreats.

Pathogen detection is typically achieved through three analytical methods: mass spectrometry, affinity-based binding, and DNA/RNA signature binding [110,111]. Mass spectrometry identifies pathogens based on molecular mass and is well-suited for multiplex detection, often employing chromatography to separate components and enhance sensitivity, along with Raman chemical imaging to provide molecular structural information [112]. Affinity- and nucleic acid-based techniques involve direct molecular interactions with biological components, yielding highly sensitive and specific detection.

Currently, enzyme-linked immunosorbent assay (ELISA) and polymerase chain reaction (PCR) remain the gold standards for pathogen detection [113]. ELISA leverages enzyme-antibody interactions to generate detectable colorimetric changes. Four ELISA types are widely used: direct, indirect, sandwich, and competitive [114,115]. While ELISA offers high sensitivity and specificity, drawbacks include the high cost of antibodies, false positives/negatives, and short shelf life due to antibody and enzyme instability [116]. PCR enables exponential amplification of target DNA sequences, providing exceptional sensitivity and specificity [112,117]. However, due to the sensitivity of PCR, contamination in samples can be highly detrimental to getting accurate results. PCR can sometimes amplify the incorrect sequence based on either the DNA polymerase or the primer, leading to false negatives [117].

**Figure 4:**
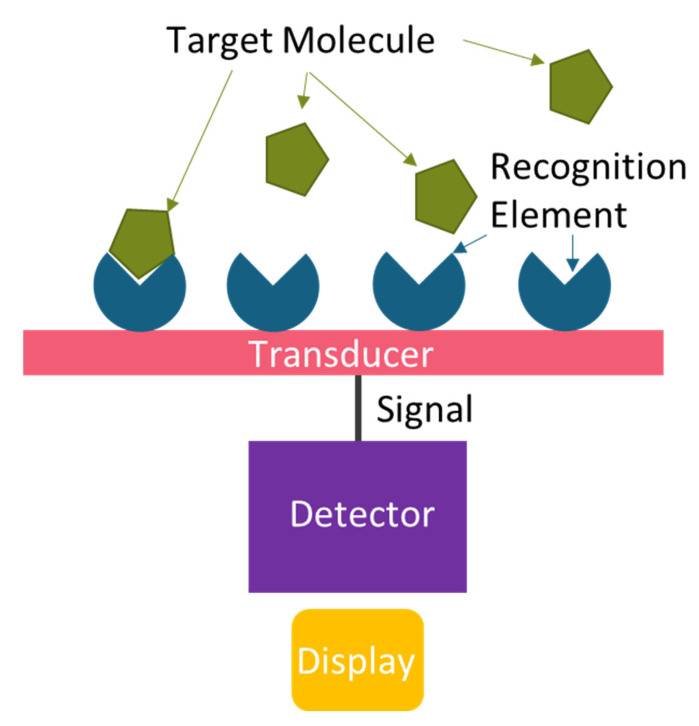
Basic Schematic of a Biosensor showing the six core components.

While ELISA and PCR provide robust detection capabilities, they are often impractical for wide-scale, rapid surveillance due to cost and complexity. Biosensors can complement these methods, offering preliminary broad screening to direct gold-standard confirmatory testing [113]. A biosensor is an analytical device that binds a target molecule and converts the interaction into a measurable signal. Biosensors typically comprise six core components: target molecule, recognition element, transducer, signal, detector, and display (Figure 4) [118,119,120,121]. All of these are necessary for the proper functioning of a biosensor; however, the recognition element and the transducer are the defining components that lead to the nomenclature for sensor types.

### 4.1. Recognition Elements

The choice of a recognition element is critical in biosensors designed for biothreat detection, as it directly determines the sensor’s specificity and reliability when identifying hazardous biological agents. Common recognition elements include antibodies, enzymes, nucleic acids, molecularly imprinted polymers (MIPs), and aptamers, each offering distinct advantages and limitations depending on the nature of the biothreat target [113,122].

Antibodies are the recognition element used in immunosensors. They are a popular candidate for biothreat detection due to their natural ability to bind specific antigens with high affinity [123]. However, antibody production is expensive and time-consuming, and their structural sensitivity to elevated temperatures, pH, and humidity significantly limits their use in rugged field conditions often associated with biothreat monitoring [122].

Enzymes, the pioneering recognition element used in biosensing [124], have been used to detect metabolic byproducts or toxins associated with biothreat agents. While they offer excellent target specificity, their catalytic activity is highly susceptible to degradation outside controlled environments, limiting their suitability for continuous or field-based applications [122,125].

Complementary Nucleic Acid binding is a strong candidate for specific detection of DNA targets. Genosensors that detect specific nucleic acid sequences can be powerful tools for identifying viral genomes or bacterial DNA, but their applicability is restricted to known genomic targets and may require extensive sample preparation [122].

Molecularly Imprinted Polymers (MIPs) offer a robust and low-cost synthetic alternative by mimicking antibody–antigen binding through templated polymerization [126]. While they are stable and can be designed for a variety of targets, their relatively low selectivity due to non-specific binding makes them less ideal for detecting low-concentration airborne biothreats in complex environments [122].

Aptamers have emerged as promising recognition elements for biosensors aimed at detecting airborne biothreats. These synthetic oligonucleotides are selected through combinatorial SELEX to bind specific targets and have been selected to bind viral proteins, bacterial toxins, or whole pathogens, with high affinity [127]. Aptamers are chemically stable, easily synthesized and modified, and maintain function across a wide range of environmental conditions, making them ideal for field-deployable devices [128,129]. Additionally, their compatibility with miniaturized electrochemical sensors enhances their utility in portable biosensing platforms. Although aptamers can suffer from non-specific adsorption, this limitation can be minimized through optimized immobilization strategies, such as those using hetero-bifunctional crosslinkers, significantly improving sensor performance and reproducibility [130]. Given their adaptability, cost-effectiveness, and robustness, aptamers are actively being investigated as recognition elements for advanced biosensor platforms targeting airborne biothreats.

To complement the discussion above, Table 4 provides a concise comparison of commonly used recognition elements in biosensors for biothreat detection. The table highlights their typical targets, key advantages, notable limitations, and relative suitability for field-deployable applications. This summary allows for quick evaluation of each recognition element’s strengths and weaknesses when designing biosensors for portable or rugged environments.

### 4.2. Transduction Mechanisms

The transducer is a critical component in any biosensor, as it converts the recognition event between the target and the recognition element into a measurable signal. Optical and electrochemical transduction modes are prominent for biothreat detection due to their sensitivity, adaptability, and potential for miniaturization and real-time analysis [131]. Other modes, such as thermal and gravimetric transduction, including Quartz Crystal Microbalance (QCM) and Surface Acoustic Wave (SAW) devices, have also been applied to pathogen detection, where they measure shifts in resonance frequency or acoustic wave propagation in response to target binding. However, these approaches are less commonly employed for field-deployable biothreat sensing due to their reliance on precise instrumentation and sensitivity to environmental [132,133].

#### 4.2.1. Optical Biosensors

Optical biosensors are among the most widely used platforms due to their versatility and adaptability across a broad range of target-recognition interactions. These sensors detect optical changes that occur upon binding between a recognition element and its target. Optical detection can be broadly categorized into labeled and label-free strategies. In labeled optical biosensing, either the recognition element, target molecule, or a complementary molecule is tagged—typically with a fluorescent label—and changes in fluorescence (such as quenching or emission) are monitored upon binding. While this approach is widely used, it presents several challenges: the labeling process is time-intensive, the label may alter the recognition/target molecule binding, and the label can detach over time, leading to instability and noise in the signal [134,135,136,137].

In contrast, label-free optical sensing offers a more streamlined and cost-effective alternative. It directly measures optical changes, such as shifts in refractive index, absorbance, or scattering, resulting from the target-recognition interaction. This approach not only reduces time and costs but also provides more direct and reliable information about the molecular interaction, without the complications associated with labeling [121,137,138]. In the context of biothreat detection, label-free optical biosensors have demonstrated impressive capabilities. For example, Sikora et al. [139] developed a label-free luminescence-based sensor capable of detecting seven different pathogenic bacteria relevant to biowarfare. Similarly, Petrovszki et al. [140] utilized evanescent field scattering to detect *Escherichia coli*, showing promise for detecting airborne or waterborne bacterial threats. Janik et al. [141] advanced label-free detection further by creating a highly sensitive optical fiber biosensor based on a microcavity in-line Mach–Zehnder interferometer (μIMZI), functionalized with peptide aptamers to detect *E. coli* O157:H7. This biosensor demonstrated excellent specificity, stability, and a detection limit of 10 CFU/mL (colony-forming units per mL. Fernández Blanco et al. [142] also reported a nanophotonic biosensor that rapidly detects *Listeria monocytogenes* in food samples using CMOS-compatible silicon nitride ring resonators, achieving high sensitivity and significant improvement in detection speed compared to traditional methods.

Building on these advances, recent research has focused on making optical biosensors field-deployable. Shen et al. [143] developed a cost-effective, chip-integrated sensing system for naked-eye visualization of PCR products, enabling rapid and portable detection of bacterial pathogens in resource-limited settings. Likewise, Jiao et al. [144] reported a field-deployable immuno-solid-phase microextraction platform coupled with portable photothermal imaging (iSPME-PI), which achieved ultralow detection limits for SARS-CoV-2 and influenza A nucleoproteins and demonstrated effective surveillance in wastewater and clinical samples. These studies highlight how optical biosensors are increasingly being adapted for on-site pathogen detection, extending their utility beyond the laboratory and into real-world biothreat monitoring.

While optical biosensors continue to show promise in biothreat detection, their use is often limited by factors such as fabrication costs, the need for complex instrumentation, and susceptibility to interference from environmental conditions [136,137,145]. Nevertheless, these sensors are widely used due to their versatility and remain a robust and promising approach for pathogen detection across diverse applications. Furthermore, the sensitivity and performance of optical biosensors can be further enhanced through the use of nanomaterials as discussed in Section 4.3.

#### 4.2.2. Electrochemical Biosensors

Electrochemical biosensors are promising candidates for biothreat detection due to their high sensitivity, rapid response, miniaturization potential, and compatibility with portable platforms [146,147,148]. Electrochemical sensors function by measuring electrical signals generated from the interaction between a recognition element and a target molecule. The transducer typically consists of a set of electrodes, where binding events lead to detectable changes in electrical properties.

There are several types of electrochemical biosensors, each monitoring different signal modalities. Potentiometric biosensors detect changes in electric potential resulting from binding at the working electrode relative to a reference electrode. Amperometric biosensors measure the current produced during redox reactions at a fixed potential, with the current magnitude proportional to the concentration of the target. Voltammetric biosensors extend this principle by varying the applied potential, allowing for multi-analyte detection. Conductometric biosensors track changes in conductance due to molecular interactions, while impedimetric biosensors—particularly relevant in recent biosensing work—measure impedance shifts in response to binding events by analyzing frequency-dependent AC signals [120,149].

Electrochemical biosensors have been widely applied in pathogen and biothreat detection. Setterington and Alocilja [150] developed a sensor integrating cyclic voltammetry with immunomagnetic separation to detect *Bacillus anthracis* and *E. coli*. Pintavirooj et al. [151] reported a voltammetric biosensor for *Klebsiella pneumoniae* using MIPs on screen-printed electrodes, achieving a practical detection limit of 10 CFU/mL. Gao et al. [152] designed a multiplex amperometric biosensor to simultaneously detect four bloodborne bacterial pathogens, including *E. coli* and *Staphylococcus aureus*, using DNA-functionalized gold electrodes. Wei et al. [153] introduced a sensor that uses magnetic separation and click chemistry to detect *Salmonella typhimurium*, achieving high sensitivity with a detection limit of 10 CFU/mL in milk samples.

Furthermore, recent research has focused on adapting electrochemical biosensors for field-deployable and wearable applications. Hannah et al. [154] demonstrated a screen-printed carbon impedance sensor capable of real-time, on-site detection of common wound pathogens, showing rapid response and robustness in complex sample matrices. Similarly, Wu et al. [155] developed a flexible “Lab-on-Cloth” electrochemical platform that integrates DNA nanorobotics with 2D graphyne. From this, they show ultra-sensitive pathogen detection in sugarcane, achieving femtomolar detection limits while maintaining portability and ease of use in field settings. Together, these studies highlight the growing feasibility of electrochemical biosensors for wearable and portable applications, supporting immediate, on-site pathogen diagnostics outside traditional laboratory settings.

Electrochemical biosensors face challenges, including susceptibility to interference from complex sample matrices, electrode fouling, limited stability of certain recognition elements, and the need for careful calibration to maintain accuracy. Despite these limitations, electrochemical biosensors are powerful tools for biothreat detection due to their high sensitivity, rapid response times, miniaturization potential, and ability to integrate with portable, real-time diagnostic platforms. These sensors can provide accurate, on-site detection of pathogens with minimal sample preparation, making them ideal for field applications. Like optical sensors, they too can be enhanced through the integration of nanomaterials, enabling greater sensitivity, improved signal clarity, and compact sensor designs suitable for real-world deployment. To provide a clear comparison of the sensors discussed above, Table 5 summarizes representative optical and electrochemical biosensors for biothreat detection, including key performance metrics, specificity, technology readiness levels (TRLs), and any nanomaterial enhancements.

### 4.3. Enhancement Using Nanomaterials

Nanostructured materials are making a significant impact on the development of biosensors, particularly in the realm of biothreat detection. The integration of nanomaterials enables sensor miniaturization, allowing for the use of smaller sample volumes and reducing overall sensor costs. Additionally, nanomaterials can improve the sensor’s signal-to-noise ratio by reducing the working electrode’s size to match the target, enhancing binding efficiency, and minimizing non-specific absorption that can introduce noise into the signal [118]. One prime example of a nanomaterial used in sensing is nanoporous anodized aluminum oxide (NAAO), which has emerged as a highly effective surface for electrochemical biosensors. NAAO is produced through electrochemical anodization of aluminum, offering a rapid and cost-effective process to create highly ordered, uniform nanopores. This structure increases the interaction area for molecular binding, boosting signal sensitivity and improving overall sensor performance [161].

Numerous studies demonstrate NAAO’s effectiveness in creating highly functional and sensitive biosensors. Jiang et al. [158] developed a label-free biosensor using NAAO functionalized with a bovine derived antimicrobial peptide (NK2A) for detecting bacterial endotoxins like Lipopolysaccharide (LPS) at a sensitivity of 10 ng/mL, demonstrating that NAAO is ideal for rapid, point-of-care diagnostics. Anisuzzaman et al. [159] utilized NAAO functionalized with a DNA aptamer specific to pyoverdine Pf5 to selectively detect *Pseudomonas protegens* activity. Banerjee et al. [12] reported an NAAO-based electrochemical aptasensor for detecting Ebola virus glycoproteins, achieving high specificity and sensitivity, with a dissociation constant (K_d_) of 2.2 nM, demonstrating its potential for early-stage Ebola detection. Similarly, Gosai et al. [160] demonstrated an NAAO-based aptamer-functionalized sensor capable of detecting human α-thrombin with a detection limit of 10 pM, even in the presence of high concentrations of interfering proteins like human serum albumin (HSA). Collectively, these examples showcase the versatility and promise of NAAO as a biosensor surface, solidifying its potential for biothreat detection and point-of-care diagnostics.

Additionally, other nanomaterials like carbon nanotubes have shown substantial promise in biosensors. Zelada-Guillén et al. [162] developed a carbon nanotube-based potentiometric aptasensor capable of detecting *Salmonella enterica* serovar Typhi and *E. coli*. Qiu et al. [156] designed a lateral flow biosensor using multi-walled carbon nanotubes functionalized with amine-modified DNA probes, achieving visual detection of DNA sequences down to 0.1 nM, with a quantitative detection limit of 40 pM. Pinals et al. [157] employed single-walled carbon nanotubes in an optical sensor to detect the SARS-CoV-2 spike protein, showing a two-fold fluorescence increase upon exposure to the spike protein.

In conclusion, incorporating nanomaterials such as carbon nanotubes and NAAO into biosensors significantly enhances their performance. These materials improve the signal-to-noise ratio, enable miniaturization, and support the development of portable, field-deployable diagnostic tools for pathogen detection, making them highly sensitive, rapid, and adaptable for detecting biothreats.

## 5. Challenges and Future Directions

The integration of microfluidic-based particle capture and biosensing technologies for detecting airborne biothreats has shown great promise but faces several significant challenges that must be overcome to realize their full potential. These challenges span technical and operational domains, each requiring targeted solutions to enable the development of efficient, reliable, and scalable systems.

### 5.1. Capture Efficiency

A pressing challenge is that of improving particle capture efficiency, especially for small, aerosolized pathogens like viruses. Microfluidic systems designed for larger particles, such as bacterial spores, have demonstrated high capture efficiencies, but these methods are less effective for smaller particles. While condensation-based growth and other amplification techniques show potential, their integration into microfluidic systems for real-time, high-efficiency pathogen capture remains complex. Moving forward, future research should aim to optimize these techniques, particularly for smaller particles, and enhance the capture efficiency for sub-micron and low-concentration aerosolized biothreats.

### 5.2. Detection Sensitivity and Specificity

Rapid detection and monitoring of biothreats requires sensing systems with high sensitivity and specificity. A key factor influencing biosensor performance is the immobilization of specific capture elements, such as aptamers, on sensor surfaces. Our previous research [130] demonstrated that hetero-bifunctional crosslinkers, like sulfo-SMCC, were significantly more effective for aptamer immobilization compared to homo-bifunctional crosslinkers (such as glutaraldehyde), leading to higher immobilization densities and more consistent sensing responses. These improvements could contribute to the overall sensitivity and specificity of biosensors designed for detecting airborne biothreats.

### 5.3. Real-Time and Continuous Monitoring

Another critical aspect is the seamless integration of microfluidic capture systems with compact biosensing platforms to achieve real-time and continuous monitoring. Many existing systems require separate components for sample collection, processing, and analysis, which can introduce delays and reduce the efficiency of real-time detection. For effective airborne biothreat monitoring, it is essential to create systems where microfluidic particle capture and biosensing are fully integrated into a single, compact device. This would enable more rapid and efficient sample handling, minimizing the need for manual intervention. Advances in microfluidic design, such as incorporating efficient aerosol capture mechanisms into small-scale, automated biosensing units, are essential for improving the speed and sensitivity of detection systems. Ultimately, the successful integration of these technologies will enable real-time, autonomous monitoring of airborne biothreats with minimal downtime, recalibration, and user input. In this regard, the potential to develop fully integrated, portable systems becomes an exciting prospect for field-deployable diagnostics.

### 5.4. Multiplexing and Broader Detection Capabilities

As real-time monitoring becomes more feasible, a significant limitation of current biosensors is their focus on detecting a single pathogen or pathogen class. For true versatility and utility in biothreat detection, future systems will need to simultaneously detect a broader range of potential threats. Electrochemical aptasensors, with their ability to incorporate multiple aptamers, offer a promising solution for this challenge. By functionalizing electrochemical sensors with a variety of pathogen-specific aptamers, it is possible to achieve multiplexing capabilities within a single microfluidic platform. However, the challenge lies in maintaining high specificity and sensitivity while enabling multiplexing without increasing system complexity. The integration of multiple aptamers and different detection principles in electrochemical biosensors could significantly expand the detection capabilities of these platforms without compromising their performance or ease of use. This ability to detect a wider array of biothreats within one compact system aligns with the growing need for highly adaptable, multi-target detection platforms.

### 5.5. Scalability and Field Deployment

In addition to expanding detection capabilities, translating laboratory-scale devices into field-deployable systems presents another critical challenge. Real-world environments, such as public spaces, military sites, and industrial areas, introduce variables like vibrations and fluctuating temperature and humidity, which can affect system performance. For successful field deployment, biosensors must be portable, robust, and low maintenance, minimizing the need for complex calibration. Achieving a compact, portable system is particularly challenging since high-end lab instruments, such as potentiostats, are often too bulky for practical field use. Even within electrochemical sensing, a key challenge lies in developing portable potentiostats, which tend to offer lower sensitivity than their lab-scale counterparts. However, electrochemical aptasensors present a promising solution, as they are not only stable across diverse environmental conditions but also compatible with compact systems. Their robustness, small size, and adaptability to different targets make aptasensors ideal candidates for field-deployable systems capable of delivering reliable performance, even with the constraints of portable potentiostats.

### 5.6. Future Research Directions

Despite the challenges, the future of airborne biothreat detection looks promising, particularly with the potential of electrochemical aptasensors and integrated microfluidic systems. Continued advancements in microfluidic designs, biosensing technologies, and particle capture methods will drive innovation in the field. Future research should focus on refining these systems’ scalability, efficiency, and sensitivity while exploring new materials, aptamer designs, and detection principles to enhance performance. Key areas of exploration include improving real-time monitoring capabilities, developing more versatile multiplexed detection platforms, and optimizing portable systems for field deployment. Computational strategies such as machine learning and artificial intelligence have shown potential to enhance biosensor signal interpretation, multiplexed detection, and automated decision-making. Future studies integrating AI/ML with portable biosensors may further improve sensitivity, specificity, and real-time analysis. Addressing these challenges will pave the way for highly reliable, portable, and cost-effective solutions capable of detecting a wide range of airborne biothreats. By combining these advancements, future research holds the potential to revolutionize biothreat detection, moving closer to practical, field-deployable diagnostic tools that are both sensitive and robust.

## 6. Conclusions

In conclusion, significant progress has been made in developing biosensor technologies for airborne biothreat detection, but overcoming the challenges limiting their large-scale deployment will be key to unlocking their full potential. The combination of microfluidic-based aerosol capture and biosensing offers a promising approach for real-time, high-efficiency detection of airborne pathogens. By addressing the challenges outlined in this review, researchers and engineers can pave the way for the next generation of devices capable of capturing and providing rapid, reliable, and scalable detection of biothreats. Such advancements will be crucial for improving global health security and ensuring timely responses to potential biothreats, ultimately contributing to public health and safety.

## Figures and Tables

**Table 1:** History of Epidemics/Pandemics [15,16,17,18].

Date (Year)	Epidemics/Pandemics	Pathogen	Death Toll (Millions)
165–180	Antonine Plague	Variola virus ^a^	5–10
541–549	Plague of Justinian	*Yersinia pestis*	15–100
735–737	Japanese smallpox	Variola virus	2
1346–1353	Black Death	*Yersinia pestis*	75–200
1519–1520	Mexico smallpox epidemic	Variola virus	5–8
1545–1548	Cocoliztli epidemic	*Salmonella enterica* ^a^	5–15
1576–1580	Cocoliztli epidemic	*Salmonella enterica* ^a^	2–2.5
1629–1631	Italian plague	*Yersinia pestis*	1
1656–1658	Naples Plague	*Yersinia pestis*	1.25
1772–1773	Persian Plague	*Yersinia pestis*	2
1846–1860	Cholera pandemic	*Vibrio cholerae*	1+
1855–1960	Third Plague Pandemic	*Yersinia pestis*	12–15
1889–1890	Flu pandemic	Influenza A/H3N8 ^a^	1
1918–1920	Spanish flu	Influenza A/H1N1	17–100
1918–1922	Russia typhus epidemic	*Rickettsia prowazekii*	2–3
1957–1958	Influenza pandemic	Influenza A/H2N2	1–4
1968–1969	Hong Kong flu	Influenza A/H3N2	1–4
1981–present	HIV/AIDS pandemic	HIV-1	44+ (as of July 2025)
2019–present	COVID-19 pandemic	SARS-CoV-2	7 (as of June 2025)

^a^ causative pathogen has not been conclusively identified.

**Table 2:** CDC-Defined Critical Biological Agents and Their Potential Use in Bioterrorism. Source: Frischknecht [24].

Category	Disease	Pathogen	Historical Abuse
A	Anthrax	*Bacillus anthracis*	World War I, World War II, Soviet Union (1979), Japan (1995), USA (2001)
	Botulism	*Clostridium botulinum*	-
	Hemorrhagic Fever	Marburg virus	Soviet bioweapons program
	-	Ebola virus	-
	-	Arenaviruses	-
	Plague	*Yersinia pestis*	Fourteenth-century Europe, World War II
	Smallpox	Variola major	Eighteenth-century North America
	Tularemia	*Francisella tularensis*	World War II
B	Brucellosis	Brucella	-
	Cholera	*Vibrio cholera*	World War II
	Encephalitis	Alphaviruses	World War II
	Food Poisoning	Salmonella, Shigella	World War II, USA (1990s)
	Glanders	*Burkholderia mallei*	World War I, World War II
	Psittacosis	*Chlamydia psittaci*	-
	Q Fever	*Coxiella burnetii*	-
	Typhus	*Rickettsia prowazekii*	World War II
	Various Toxic Syndromes	Various bacteria	World War II
C	Emerging pathogens		

**Table 3:** Comparison between various microfluidic-based capture systems grouped by operating mechanism.

Operating Mechanism	Microfluidic Design	Target Particle/Organism	Advantages/Limitations
Inertia-based/Passive mixing	Staggered herringbone microchannels [45,46,47,48,49]	*E. coli*, *M. smegmatis*, *M. tuberculosis*	Simple design, efficient for bacteria; longer capture times (1–3 h)
	Herringbone microchannels [50]	*Aspergillus niger* spores	Good for spores; limited real-time sensitivity
	Three-loop spiral with herringbone & sawtooth [51]	General aerosol particles	Improved mixing; manual steps required
	On-chip impinger [54]	Microorganisms	Moderate throughput; simple operation
	Inertial forces + mini fluorescent microscopy [55]	Microorganisms	Real-time detection; limited scale
	Droplet-based + wet-cyclone sampler [56]	Particles 2–5 µm	Captures medium particles; poor submicron efficiency
	Size-based separation microfluidic system [57]	Submicron particles	Size-selective; complex operation, ~70% efficiency
	U-shaped stratified liquid stream [60]	Particles 0.6–2.1 µm	High submicron efficiency; more complex channel design
	U-shaped stratified flow microchannel [11,44]	Aerosol particles	Validated numerically & experimentally; improved capture
Electrostatic	Electrostatic microfluidic sampler [58]	Particles < 5 µm	Can target small particles; may alter biological properties, ~40% efficiency
Filtration-based	PDMS microfilter-based membrane [48]	General bioaerosols	Simple and passive; low throughput, time-intensive
Sensor-based/Electrical	Silicon nanowire FET + microfluidic [53]	Influenza virus	Enables electrical detection; limited sensitivity (20–30% signal increase)
Integrated/Multi-step	Semi-automated microfluidic chip [52]	Spores	Combines collection & amplification; multi-step, moderate throughput

**Table 4:** Summary of common biosensor recognition elements, their typical targets, advantages, limitations, and field-deployability.

Recognition Element	Typical Targets	Advantages	Limitations	Field-Deployability
Antibodies	Proteins, toxins, pathogens	High specificity and affinity; well-established	Expensive; sensitive to temperature, pH, humidity	Low–Moderate
Enzymes	Toxins, metabolites, small molecules	Rapid signal; high catalytic specificity	Short shelf life; denatures easily	Low
DNA/RNA Probes	Nucleic Acid Sequences	Sequence-specific; strong specificity	Limited to known nucleic acid targets; cannot detect whole pathogens	Moderate
Aptamers	Proteins, toxins, pathogens, small molecules	Chemically stable; easily modified; high affinity; suitable for portable devices	SELEX can be slow; some non-specific adsorption	High
Molecularly Imprinted Polymers (MIPs)	Small molecules, proteins, toxins	Synthetic and robust; long shelf life; inexpensive	Lower selectivity; less sensitive in complex samples	High

**Table 5:** Comparison of optical and electrochemical biosensors for biothreat detection. The table summarizes target analytes, performance (limit of detection and response time), specificity, and any nanomaterial enhancements.

Author	Sensor Type	Target(s)	Performance (LoD/Time)	Specificity	Nanomaterial
Sikora et al. [139]	Optical	Multiple bacteria	10^2^–10^6^ CFU/mL/Real-time	High	None
Petrovszki et al. [140]	Optical	*E. coli*	~10^2^ CFU/mL/N/A	High	None
Janik et al. [141]	Optical	*E. coli* O157:H7	10 CFU/mL/N/A	High	None
Fernández Blanco et al. [142]	Optical	*Listeria monocytogenes*	10^2^ CFU/mL/4 h	High	None
Shen et al. [143]	Optical	*E. coli*, *S. enterica*	3 copies/Few hours	High	None
Jiao et al. [144]	Optical	SARS-CoV-2 N, Flu A NP	68–75 pg/mL/15 min	High	None
Qiu et al. [156]	Optical	Specific DNA sequences	40 pM/min	High	Multi-walled carbon nanotubes (MWCNTs)
Pinals et al. [157]	Optical	SARS-CoV-2 spike protein	12.6 nM/Minutes	High	Single-walled carbon nanotubes (SWCNTs)
Setterington & Alocilja [150]	Electrochemical	*Bacillus cereus*, *E. coli* O157:H7	40 CFU/mL/65 min (*B. cereus*); 6 CFU/mL/65 min (*E. coli*)	Moderate	None
Pintavirooj et al. [151]	Electrochemical	*Klebsiella pneumoniae*	0.012 CFU/mL/N/A	High	None
Gao et al. [152]	Electrochemical	Bloodstream bacteria (16S rRNA)	290 CFU/mL/1 h	High	None
Wei et al. [153]	Electrochemical	*Salmonella typhimurium*	10 CFU/mL/N/A	High	None
Hannah et al. [154]	Electrochemical	*Proteus mirabilis*, *Pseudomonas aeruginosa*, *Staphylococcus aureus*	7.4 × 10^6^ CFU/mL/1–2.5 h	Moderate	None
Wu et al. [155]	Electrochemical	Pokkah boeng pathogen (sugarcane)	16.6 aM/N/A	High	None
Jiang et al. [158]	Electrochemical	LPS (Gram-negative), LTA (Gram-positive)	10 ng/mL/N/A	High	NAAO
Anisuzzaman et al. [159]	Electrochemical	Pyoverdine Pf5 (Pseudomonas)	1.3 nM/N/A	High	NAAO
Banerjee et al. [12]	Electrochemical	Ebola virus sGP, GP1,2	150 pM/N/A	High	NAAO
Gosai et al. [160]	Electrochemical	α-Thrombin	10 pM/N/A	High	NAAO

## Data Availability

Not applicable.
